# An intelligent spam detection framework using fusion of spammer behavior and linguistic

**DOI:** 10.1371/journal.pone.0313628

**Published:** 2025-02-06

**Authors:** Amna Iqbal, Muhammad Younas, Muhammad Kashif Hanif, Muhammad Murad, Rabia Saleem, Muhammad Aater Javed

**Affiliations:** 1 Department of Computer Science, Government College University Faisalabad, Faisalabad, Punjab, Pakistan; 2 Department of Information Technology, Government College University Faisalabad, Faisalabad, Punjab, Pakistan; University of Sargodha, PAKISTAN

## Abstract

The diverse types of fake text generation practices by spammer make spam detection challenging. Existing works use manually designed discrete textual or behavior features, which cannot capture complex global semantics of text and reviews. Some studies use limited features while neglecting other significant features. However, in case of a large number of features set, the selection of all features leads to overfitting the model and expensive computation. The problem statement of this research paper revolves around addressing challenges concerning feature selection and evolving spammer behavior and linguistic features, with the goal of devising an efficient model for spam detection. The primary objective of this endeavor was to identify the most efficacious subset of features and patterns for the task of spam detection. Spammer behavior features and linguistic features often exhibit complex relationships that influence the nature of spam reviews. The unified representation of features is another challenging task in spam detection. Various deep learning approaches have been proposed for spam detection and classification but these methods are specialized in extracting the features but lack to capture feature dependencies effectively with other features but there is a lack of comprehensive models that integrate linguistic and behavioral features to improve the accuracy of spam detection. The proposed spam detection framework SD-FSL-CLSTM used the fusion of spammer behavior features and linguistic features which automatically detect and classify the spam reviews. Fusion enables the proposed model to automatically learn the interactions between the features during the training process, allowing it to capture complex relationships and make predictions based on both types of features. SD-FSL-CLSTM framework apparently shows the promising result by obtaining a minimum accuracy 97%.

## Introduction

The World Wide Web (WWW) is widely regarded as the dominant communication platform in contemporary society. Through the utilization of e-Commerce platforms, online forums, and personal blogs, individuals are able to efficiently express their opinions regarding various products or services. Online user comments, which are utilized by both customers and businesses, have gained significant recognition and importance in the realm of the internet. Vendors can utilize these evaluations for strategic planning of future manufacturing or marketing initiatives [1]. They also engage in problem-solving activities to address the issue [2].

Spam attacks are becoming more common because anyone can write and post spam content online without restrictions. Anyone can do this, and the people who do it are called spammers. Spammers offered their services to write fake comments of a business’s goods and services. Most spam comments are written to make money or promote a product or service. This behavior is called spamming [2–4]. Major commercial online web sites like Yelp [5] and Amazon [6] have already made some progress in identifying spam [7]. Systems that use real-world datasets to look for spam have a long way to go before they reach their full potential [7].

Recent techniques like machine learning are particularly effective in identifying and removing spam comments. However, spam filtering depends on the number and type of features machine learning uses for training algorithms. Feature weightage, feature identification, and selection are critical factors that impact machine learning-based spam filtering efficiency [8]. Moreover, automated product ranking systems and recommender systems are also affected by spamming [9].

One of the most challenging problems to tackle in text classification [10] is how to reduce noise from a large number of text documents, for instance, by deleting characteristics that are not necessary or are used several times. Consequently, feature extraction and selection techniques have been offered to solve this problem.

The Dimensionality Reduction (DR) approach [10] aims to increase the efficiency of transfer learning by reducing the distance or gap between the distributions of several data sets inside a latent space. The findings indicate that dimensionality reduction (DR) results are much superior to those obtained without DR [11]. With a dimensionality reduction, less processing time is required, efficiency is improved, and accuracy is improved. The two basic categories of DR approaches are feature selection (FS) and feature extraction. These are the two most usually used DR methods [12]. Due to the increasing pace of data production, feature selection (FS) has become a crucial strategy. Feature selection improves some significant dimensionality, including minimizing repetition, eliminating extraneous data, and enhancing the comprehension of findings. Finding the most distinctive, insightful, and condensed collection of features to improve the efficiency of data processing and storage is an open issue for the text classification challenge addressed by feature extraction (FE).

The feature selection strategy aims to reduce the number of features while maintaining the projected accuracy of a classifier [13]. In this manner, representative subsets of the original feature set are selected based on the significance of each representative. In data mining, deep learning is popularly used, employing algorithms to uncover and remove general principles from extremely large data sets. These algorithms could determine what the user likes on their own. In this work, two algorithms are evaluated to improve the accuracy of feature selection, including XGB [14] and PCA [15].

The existing research on spam detection primarily focuses on linguistic or behavioral methods separately. However, there is a lack of comprehensive models that integrate linguistic and behavioral features to improve the accuracy of spam detection in machine learning [9]. Additionally, most studies use a single classifier to train their models, neglecting the evaluation of multiple classifiers’ performance.

Therefore, there is a need for a research study that addresses these limitations and develops a linguistic model incorporating various linguistic features and behavioral features to enhance the accuracy of spam detection. Furthermore, evaluating the performance of different classifiers, such as Naive Bayes, Logistic Regression, Support Vector Machine, and Random Forest, would provide insights into the effectiveness of different classification approaches for spam detection.

The state-of-the-art work has been done by [9], for the spam detection in this research author focused on thirteen features while neglecting other significant features that could enhance classification accuracy. Moreover, they did not incorporate data reduction techniques, which can result in problems related to overfitting. Furthermore, the work in [9] did not explore the impact of varying the training and testing ratios on the efficiency of spam detection using deep learning models. This investigation could potentially enhance the performance of the classification model by optimizing the utilization of training and testing data. Furthermore, the overall accuracy achieved in [9] was reported as 84%, which is relatively low and indicates room for improvement in the classification model’s performance. Therefore, there is a need to find a solution for accurately selecting the best feature set for training the data model, aiming to enhance the accuracy and overall performance of spam detection. By bridging this research gap, this research makes the following contributions:

1. Spammer behavior features calculation; these thirty-three derived features contribute to spam detection.
2. A smart approach is introduced for selecting the best features sets of spammer behavior features for spam detection by applying the dimensionality reduction technique.
3. Propose a customized LSTM-based CNN (CLSTM) deep learning approach for detecting spam called SD-FSL-CLSTM framework that fused spammer behavior and linguistic features with promising accuracy.

All the above contributions are testified by comparison of various machine learning and deep learning techniques previously presented in this domain. The promising results of the proposed method ensure the novelty of the work. The novelty of this research lies in introducing the SD-FSL-CLSTM model, which combines spammer behavior and linguistic features to offer an innovative approach for effective spam review detection and improved performance. This integration allows the model to capture complex relationships and interactions between the two types of features, enhancing the accuracy of text classification. The model uses Principal Component Analysis (PCA) and XGBoost (XGB) for feature selection, identifying key linguistic features and optimizing model performance. Additionally, the calculation of spammer behavior features plays a critical role in detecting patterns associated with spam behavior, such as frequency of reviews or unusual reviewing habits. By addressing limitations in existing methodologies and providing a comprehensive solution, the proposed model demonstrates a promising step forward in the field of spam detection.

## Literature

In the domain of spam detection, scholars encounter several intricate challenges necessitating thorough investigation. These challenges encompass managing the behavioral features of spammers, scrutinizing linguistic patterns, and handling extensive feature sets, as well as evaluating large datasets. Additionally, some researchers concentrate on developing machine learning models tailored to spam detection, which poses distinct challenges due to the evolving tactics of spammers. Addressing these pivotal research areas can significantly improve the accuracy and applicability of machine learning models in a variety of practical contexts.

### Feature reduction techniques

XGB is a popularly known algorithm for the achievement of higher accuracy and overcomes overfitting the model [16]. XGB offers the following advantages: (1) Good handling of missing data; (2) Minimizing overfitting, and (3) Lowering running time by combining parallel and distributed processing. Regarding accuracy and overfitting, the XGB setup with a maximum depth of 10, a learning rate of 0.3, and more than 100 iterations exhibit better learning accuracy in the work done by [17]. XGB based approach is more precise than conventional logistic regression prediction [18], principal component analysis (PCA) [19], linear discriminant analysis (LDA) [20] and other feature extraction methods using low-dimensional feature space instead of original feature space. A tiny subset of the initial collection of characteristics may be chosen using feature selection strategies based on the relative importance of each feature. Even though wrapper models can generate different feature sets, even though they use a specific classifier to evaluate and select features, even though they can generate different feature sets [14], and even though they can find a better and non-redundant feature set by the classifier with cross-validation [21, 22].

In contrast to wrapper models, filter models use various evaluation methods rather than classifiers [23]. Also, because they process data rapidly and effectively, filter models are often used to scale huge datasets. Classifiers are used in wrapper models. Feature-ranking approaches attempt to rank features in terms of importance within filter models using various criteria [31]. Many strategies that are comparable to this one has been used to choose characteristics for text categorization [24]. Document frequency (DF), term frequency (TF), and document term frequency (DTF) are three categories into which these techniques might be divided (DTF). Many ideas, including term frequency, information gain [25], and chi-square [26], have been proposed within these three groupings (TF). Members of the DF family include CHI, IG, GINI, IMGI, and DF [27, 28]. Members of the TF and TFIG families have TTFS and TFIG. Members of the DTF family include TFIDF and IMTFIDF.

### Spammer behavior features

In this particular section, the research investigates and explained the distinct characteristics and attributes linked to the behavior of spammers. The study [29] described that the spammer detection technique based on user behavior helps to locate spam and identify its nature. A model that employs the reviewer’s time series properties was proposed by [30]. Then, a genuine Amazon dataset was used to test this model. A text mining model that was based on the integration of time across many time periods and employed an unsupervised technique and features were presented by [29]. Additionally, this model integrated a semantic language model designed to detect spam with a dataset from Yelp. [31] They developed an algorithm to identify that were published with malicious intent after discovering a connection between people and products in their investigation. The study [32] presented an interaction network-based behavior detection framework based on a graph structure known as SEINE (Spam Detection Using Interaction Networks). [33] Presented a classification system called EUPHORIA that can distinguish between spam and legitimate text. EUPHORIA blends multiview learning with deep learning to increase accuracy. The recommended approach achieved a maximum AUC-ROC of 0.813. The study [34] developed a neural network model that employs a pre-trained BERT language model to acquire details about the message context and recognize spam using only content-based characteristics, both generic spam, and spam that is particular to a certain context [35]. The neural network model outperforms with an F1 score of 0.91 and handled the dataset imbalance too. Author [36] proposed a deep feature fusion approach that strikes a balance between the importance of textual and behavioral information. Most of the research [34, 36] on spammer behavior exclusively employed time series-based spammer behavior characteristics, according to a spammer behavior models. It may be easier to identify spammers if a broad range of behavioral attributes are used. Many researchers utilized the Yelp Reviews [37] and TripAdvisor [38] datasets. Most of the behavioral framework makes use of multiple use characteristics of spammers’ behavior showed in Table 1 to calculate the spam score in the context of spam detection

**Table 1:** Spammer behavior used in literature.

	F1	F 2	F 3	F 4	F 5	F 6	F 7	F 8	F 9	F 10	F 11	F 12	F 13	F 14	F 15	F 16	F 17	F 18	F 19	F 20	F 21	F 22	F 23	F 24	F 25	F 26	F 27	F 28	F 29	F 30	F 31	F 32	F 33
[39]	✓	✓											✓			✓								✓	✓	✓	✓	✓	✓	✓	✓	✓	✓
[40]	✓		✓	✓							✓	✓	✓	✓	✓			✓															
[41]	✓	✓										✓	✓	✓			✓																
[42]					✓	✓	✓		✓									✓	✓	✓	✓	✓	✓										
[9]	✓	✓		✓	✓			✓				✓	✓	✓	✓		✓			✓									✓		✓		
[43]	✓						✓							✓			✓																
[44]	✓	✓			✓					✓										✓													
[45]	✓				✓							✓		✓			✓																
[46]	✓										✓	✓					✓																

### Spam detection using linguistics features

In this particular section, the research investigates and explained the distinct characteristics and attributes linked to the Linguistics. The first study [39] examining the challenge of identifying fake comments was conducted in 2007. The study involved evaluating 5.8 million customer comments on Amazon.com, an e-commerce platform. The researchers focused solely on the text. During the course of their investigation, they discovered numerous duplicated texts and concluded that spammers frequently recycle content, albeit with slight modifications. The authors used the logistic regression classifier to teach the model how to work. this work Showed [40] that the semantic language model has been used to find spam. The authors used a classifier based on the Support Vector Machine to train their suggested technique. [41] Used a supervised learning strategy and a co-training method to find spammers according to their language patterns. The author suggested a way to group things that used N-gram characters as a linguistic trait [47, 48]. The Naive Bayes classifier was also used in the suggested strategy to tell the difference between spam and real comments. Study [42] used statistically based features for the Extreme Gradient Boost Model and the Generalized Boosted Regression Model to analyze datasets with more than one language according to the experiment results, the Generalized Boosted Regression Model proved to be more effective for the Malay dataset, while the Extreme Gradient Boost Model was more successful for the online dataset. The author [44] showed how to use supervised learning hierarchically. The study [49, 50] finds spam comments using a supervised model based on reviewer characteristics. The authors gave several time-sensitive characteristics to ensure that spam comments could be found as soon as possible. They then used an SVM classifier to train the model. The study [51] used the feature-based sparse additive generative model and the SVM classifier to find the general rule for spotting spam comments.

Like DNN-based methods for botnet detection, such as Dnnbot [52], which combines deep learning with network traffic analysis the purposed SD-FSL-CLSTM model integrates both linguistic and spammer behavior features for improved classification accuracy in spam detection. The growing use of blockchain frameworks, particularly for secure online data management​ suggests potential applications in spam detection where immutable records could help track spammer behavior and reduce fraudulent reviews. In addition to spam detection, deep learning models like DBoTPM [53] have proven effective in identifying botnets, further demonstrating the versatility of neural networks in cybersecurity contexts. Target detection frameworks utilizing CNNs [48] highlight the importance of deep learning for pattern recognition, an approach mirrored in our use of LSTM-CNN for detecting spam patterns in text. Supervised learning models have been successfully applied to text classification, underscoring the effectiveness of combining linguistic features with spammer behavior data for spam detection. In their work, [54] explored spam detection in Amazon reviews using advanced machine learning algorithms and achieved significant results. We have now included a direct comparison between their approach and our proposed SD-FSL-CLSTM model. Specifically, this research focused on extracting specific features from Amazon review text, our approach combines both linguistic and spammer behavior features, providing a more comprehensive detection framework. The author reported an accuracy of 93%, while our SD-FSL-CLSTM model achieved a higher accuracy of 97.57%. This improvement can be attributed to the integration of behavioral features with linguistic data, enhancing the model’s ability to detect complex spam patterns.

Our proposed SD-FSL-CLSTM model achieved an accuracy of 97.57%, which slightly outperforms [55] model. While their method integrates RL, which allows for dynamic feature selection and adjustment based on feedback, our model benefits from the fusion of linguistic and behavioral features, combined with PCA and XGBoost, which enhances both feature selection and classification accuracy. The author used reinforcement learning and introduces a layer of adaptability that is not present in our method, but our focus on feature optimization using PCA and XGBoost allows us to capture more complex relationships between features, which likely contributes to the higher performance in our model.

Previous studies [9, 40, 51, 56] did not take into account several linguistic features when constructing SRD models, relying instead on a single classifier to train their models. This research work extends the scope of SRD research by creating a linguistic model that incorporates various feat0ures such as stemming and N-gram, and word2vec techniques. These modifications have significantly improved the accuracy of the proposed model in detecting spam. The key findings and approaches from literature review provided a concise overview of these studies and contributed to the understanding of spam detection. XGBoost (XGB) stands out for its robust handling of missing data and minimizing overfitting, surpassing traditional methods like logistic regression and PCA. Feature selection strategies, including wrapper and filter models, address the challenge of selecting relevant features from large datasets. Integrating linguistic and behavioral features enhances spam detection accuracy, emphasizing the importance of a broad range of attributes in identifying spammers. Advanced techniques like stemming, N-gram analysis, and word2vec improve the efficacy of linguistic feature analysis. These findings collectively advocate for holistic approaches in spam detection, bridging the gap between linguistic and behavioral insights for more accurate detection methods.

## Dataset for experimentation

The dataset utilized in this study was obtained from the research work [9], providing a comprehensive collection of both spam and legitimate reviews. This dataset facilitates the investigation of various spam detection methodologies.

This study uses a data set of real Amazon product reviews [9] containing 3.1 million products, 26.7 million reviews, and 15.4 million reviewers in the data set. The data set comprises many parts, such as categories, reviewers, goods, reviews, etc. as shown in Table 2. Before training the model using the labeled dataset, the data preprocessing, tokenization, content analysis, feature extraction, selection, and classification are carried out using the Natural Language Toolkit (NLTK) library. Sourced from Kaggle [9], the dataset underpins the research, acknowledging potential biases and limitations inherent in its composition. Emphasizing the necessity of discussing dataset representativeness, factors like class distribution and relevance to the research context are considered. Transparency regarding dataset origin and characteristics strengthens the validity and applicability of findings. The analysis also performed on YelpChi [55].

**Table 2:** Amazon dataset used in the proposed framework.

Category	Total Reviews	Total Reviewers	Total Product
Cell Phones and Accessories	3446396	2260636	319652
Clothing, Shoes, and Jewelry	5748260	3116944	1135948
Electronics	7820765	4200520	475910
Home and Kitchen	4252723	2511106	410221
Sports and Outdoor	3267538	1989985	478846
Toys and Games	2251775	1342419	327653
Total	2,67,87,457	1,54,21,610	31,48,230

## Methodology

**Fig 1:**
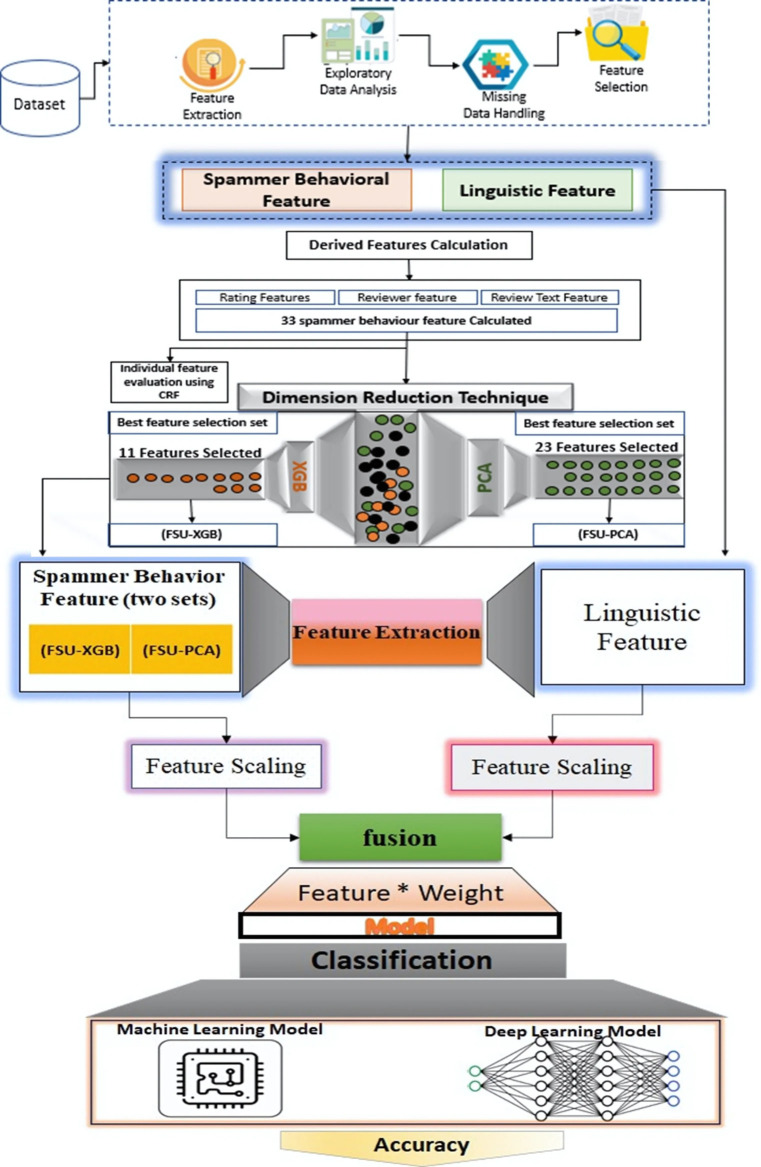
SD-FSL-CLSTM spam detection framework.

In Fig 1 intelligent spam detection framework consists of three correlated phases. In the first stage, the thirty-three derived features are calculated due to their demonstrated relevance and effectiveness in improving the model’s performance on the dataset these features are showed in Table 3 and the notations are used in this methodology is listed in Table 4.The methodology of this research paper entails several sequential steps to develop an effective spam detection model. Initially, thirty-three features are computed from the dataset, capturing various aspects of spammer behavior and linguistic characteristics. Subsequently, feature selection techniques, specifically Principal Component Analysis (PCA) and Extreme Gradient Boosting (XGB), are employed to identify the most influential features among the computed set. Following this, the selected features undergo feature scaling to ensure uniformity in their magnitudes, facilitating better model convergence. A fusion matrix is then generated by integrating linguistic features with the selected ones, aiming to capture a comprehensive representation of the text data. Finally, the fusion matrix serves as input to train the classification model, thereby enabling effective spam review detection with improved performance.

**Table 3:** Derived feature.

Id	Feature name
F-1	Rating Deviation (RD)
F-2	Percentage of Positive Reviews (PR)
F-3	Rating Abuse (RA)
F-4	Extreme Rating (EXT)
F-5	Time Series (TS)
F-6	Appending Time (AT)
F-7	User Activity (UA)
F-8	Number of reviews per day (NRPD)
F-9	Review of a single product (RSP)
F-10	User tenure (UT)
F-11	Early Time Frame (ETF)
F-12	Content similarity (CS):(reviewer)
F-13	Maximum Number of Reviews (MNR):(per-day)
F-14	Activity Window (AW)
F-15	Ratio of First Reviews (RFR)
F-16	Number of helpful feedbacks (NHF)
F-17	Review Posting/ Count (RP/C)
F-18	Content Similarity (CS)
F-19	Self-Reference (SR)
F-20	Review length (RL)
F-21	Ratio of Opinion Words (ROW)
F-22	Transition Words of the Sentiment Expression (TWSE)
F-23	Exclamatory Tone (ET)
F-24	Reviewer gives (good, bad, average sentiment) (GBAS)
F-25	Reviewer gives (good and average sentiment) (GAS)
F-26	Reviewer gives (bad and average sentiment) (BAS)
F-27	Reviewers give (good and bad sentiment) (GBS)
F-28	Percentage of positive opinion words (PPOW)
F-29	Percentage of negative opinion words (PNOW)
F-30	Percentage of numeric used (PN)
F-31	Percentage of Capital words (PCW)
F-32	All capital word used in text (ACW)
F-33	Name of the brand mentioned (NBM)

**Table 4:** Notation used in methodology.

R	Reviewer
r	Review
*T*_*r*_	Total number of reviews
*P*	Products
*NS*	Not spam
*S*	Present the spam
*W*	All reviews of reviewer R
0	Not Spam
1	Spam
SD-FSL-CLSTM	Spam Detection using Fusion of Spammer and Linguistic Feature-CLSTM

### Feature derived

The calculation and discussion of features are addressed. Sequentially, in the order they are presented in the following section.

**Rating Deviation (RD):** A rational user is anticipated to provide a rating that aligns with the rating given by another reviewer for a comparable product. The product’s mean rating value is determined using Eq 1. Next, the normalized score, also known as the rating deviation, is calculated using the mean value according to Eq 2.


$${MEAN}_{r}=\frac{\sum_{x==1}^{\left|w_{r}\right|}\left|*r_{p}\right|}{w_{r}}$$ (1)

$$F_{DR}=\frac{\left|*r_{p}-{MEAN}_{r}\right|}{4}$$ (2)

**Percentage of Positive Reviews (PR):**PR describes the ratio of positive to negative reviews. if a user tends to review positive only, he is more likely to be a spammer. A threshold limit of at least 15% negative comments is applied to judge the legitimacy of a user.PR describes the ratio of positive to negative reviews. if a user tends to review positive only, he is more likely to be a spammer PR is calculated using the Eq 3. Here NPR = Count of reviews with a positive sentiment. ***Tr*** Presents the total number of reviews.


$$PR=\left\{ \begin{array}{ll} 0, & \mathrm{if}\frac{NPR}{Tr}\times 100\geq \mathrm{Threshold}\\ 1, & \mathrm{if}\frac{NPR}{Tr}\times 100<\mathrm{Threshold} \end{array}\right.$$ (3)

**Rating Abuse (RA):** Typically, a legitimate individual would be expected to assign a score that aligns with the scores given by another individual for the same item. Spammers aim to create a misleading impression of a product, which can be achieved through positive or negative means. Spammers’ ratings consistently differ from those of genuine users. This study calculates the mean rating of the product based on a specific attribute. To compute the Rating Abuse (RA) using a given threshold (e.g., 4 or 5) on a dataset. The Eq 4. Calculate the percentage of reviews that fall below the specified rating threshold, indicating the proportion of reviews that considered as rating abuse. Here NRR presents the Number of Reviews with Rating.


$$RA=\frac{NRR<Threshold}{Tr}\times 100$$ (4)

**Extreme Rating (EXT):** Extreme rating refers to the act of assigning the highest possible score, such as 1/5 or 5/5. Users who consistently provide comments that are either the maximum or minimum value are more likely to be classified as spammers. To compute the Extreme Rating (EXT) using a given threshold (e.g., 1 or 5) from the dataset, it is necessary to ascertain the proportion of reviews with the lowest and highest ratings in relation to the total number of reviews. The Eq 5 for calculating the EXT score. Number of EC refers to the count of extreme comments.


$$EXT=\frac{Number\;of\;EC<Threshold}{T_{r}}\times 100$$ (5)

Number of extreme comments: Count of comments with the lowest = 1 and highest = 5 Ratings.

**Time Series (TS):** describes the activity of user for how long he been active on the website. For a user with only a single and long-time frame for review activity is considered as spammer compared to a reviewer who visits the site time to time and post review. The thresh hold for the Eq 6 is 30 days. Here T_end_ the latest review activity for a user.

T_start_ The earliest review activity for a user.


$$TS=\left\{ \begin{array}{l} T_{end}-T_{start},\;if\;T_{end}-T_{start}\geq T_{Threshhold}\\ 0,\;otherwise \end{array}\right.$$ (6)

**User Activity (UA):** User activity is considered to be legitimate by his user ID. Similar content from more than 5(C = 5) IDs is considered spam. On the other than, an ID is considered legitimate to review below threshold limit. The thresholds are tested using the reviewer ID showed in Eq 7.


$$UA=\left\{ \begin{array}{l} 0,\;if\;UA\leq c\\ 1,\;otherwise \end{array}\right.$$ (7)

**Maximum Number of Reviews Per Day (MNR): Posting** multiple reviews in a single day can be seen as a sign of deviant behavior. This indicator quantifies the reviewer’s maximum daily review count, normalized by the overall maximum value in our dataset Eq 8 is used to determine it. Here *MR*(*R*_*i*_) is the maximum number of reviews posted by reviewer *R*_*i*_ in a single day.


$$F_{MNR}(R_{i})=\frac{MR(R_{i})}{M_{R_{i}\in R_{i}(T_{r})}MR(R_{i})}$$ (8)

**Review Of a Single Product (RSP):** If a reviewer posts multiple reviews about the same product, it can be indicative of spam behavior.


$${User}_{RSP}=r,where\;r_{P}\in R(P)$$ (9)

In this Eq 9, r_P_ represents a review specifically related to the product P. The notation "r_P_ ∈ R(P)" indicates that the review r belongs to the set of reviews written by reviewer R for the product P. A threshold limit is applied for reviewer of single product with not more than 3 reviews to consider genuineness of user. Here c = 3.


$$RSP=\left\{ \begin{array}{l} 0,\;if\;{User}_{RSP}\leq c\\ 1,\;otherwise \end{array}\right.$$ (10)

**Early Time Frame (ETF):** Early time frame describes the time frame between the posting of product and posting of comments. An immediate review after the posting of product is more prone to be spam showed in Eq 11. Here *L R*_*i*_*(r*_*j*_*)* presents the Last date of the report authored by reviewer R_i_.


$$ETF(R_{i})=\left\{ \begin{array}{l} 0\;L(R_{i}(r)-f(R_{i}(r))>ŕ\\ \frac{L(R_{i}(r))-f(R_{i}(r))}{ŕ}\;Otherwise \end{array}\right.$$ (11)

**Content Similarity (CS):** Cosine similarity used to measure the textual or semantic similarity between pairs of reviews. Spammers often opt to copy reviews from similar products due to the time-consuming nature of generating new reviews. Therefore, it is advantageous to employ cosine similarity to identify the similarity in content between reviews written by the same reviewer. To identify the most undesirable behavior of spammers, in this research the maximum similarity approach employed the equation for the maximum similarity approach defined in following Eq 12.


$$F_{CS}=Max(Cosine(R_{i}(r_{j}),R_{i}(r_{k})))where\;R_{i}(r_{j}),R_{i}(r_{k})\in R_{i}(T_{r})$$ (12)

In the Eq 12, R_i_(r_j_) and R_i_(r_k_) represent two reviews written by reviewer Ri from the set of reviews R_i_(T_r_). The cosine similarity between R_i_(r_j_) and R_i_(r_k_) is computed using a cosine similarity function.

**Ratio Of First Reviews (RFR):** People tend to rely on the initial reviews in order to benefit from them. Spammers create email accounts early on to impact initial sales. Spammers believe that controlling initial product reviews gives them the ability to manipulate public opinion. The ratio between the initial reviews and the total reviews for each author is calculated. The term "first reviews" refers to the initial evaluations of a product that are posted by the author showing in the Eq 13. *R*_*i*_(*r*_*first*_) Represent the first review of a reviewer.


$$RFR=\frac{\left|R_{i}(r_{first}){\in R}_{i}(Tr)\right|}{R_{i}(Tr)}$$ (13)

**Review Posting/ Count (RP/C):** Review count describes the number of comments posted by a single user on the site. The count is considered as threshold to determine the spamming activity. A review count of more than 5 (obtained mean value after an experiment) is considered as spam in Eq 14.


$$\mathrm{RP}/C=\left\{ \begin{array}{c} 0,\;if\;Review\_Count\leq T\\ 1,\;if\;Review\_Count>T \end{array}\right.$$ (14)

**Self-Reference:** in reviews refers to the use of first-person language by reviewers to convey their person al experiences with a product. This emphasizes the credibility of their account. Reviews using second-person references like "you" to guide or recommend to other consumers may raise suspicions. Self-reference is important in identifying deceptive comment. Let *R* be a review, *SR*(*R*) represent the self-reference score of review *R*, Count_first-person_ (*R*) denote the count of first-person pronouns in review *R*, and Total_pronouns_ (*R*) denote the total number of pronouns in review *R*. The self-reference score *SR*(*R*) can be calculated as in Eq 15

$$SR(R)=\frac{{Count}_{first-person}(R)}{{Total}_{pronouns}(R)}$$ (15)

This score quantifies the extent to which a review employs first-person pronouns to express personal experiences, which help in identifying the use of self-reference in reviews. A threshold limit is applied to determine self-references as spam indicator. This identified (C = 3) as limit to self-reference.

**Review length (RL):** Spammers don’t have a lot to say about actual events since they strive to fabricate false experiences. You might even assert that spammers often spare little thought to composing a single review. The reviews in the dataset generally contain around 400 characters written by reviewers. This figure is used as a cutoff in the approach that was recommended to flag as spam any reviews with a total of less than X characters which is expressed in Eq 16.


$$R_{IsSpam}=\left\{ \begin{array}{l} 1,\;if\;Length(R)<X\\ 0,\;otherwise \end{array}\right.$$ (16)

**Ratio of Opinion Words (ROW):** Opinion words are linguistic expressions that play a crucial role in determining the sentiment or emotion conveyed by an individual. An individual typically expresses their opinion succinctly rather than using an excessive amount of subjective language. A threshold limit of c = 4 is utilized to assess the credibility of the review showed in Eq 17. Here *OW* presents Opinion Words.


$$R_{\mathrm{ROW}}=\left\{ \begin{array}{l} 0,\;if\;OW\geq c\\ 1,\;otherwise \end{array}\right.$$ (17)

**Transition Words of the Sentiment Expression (TWSE):** Transition words are used in writing to show the connection between ideas, particularly when expressing cause-and-effect relationships. Transition words in sentiment analysis offer valuable insights into an individual’s expression of sentiment. Normal users typically employ transition words in a natural and moderate fashion to express their viewpoints. Spammers may excessively employ transition words to deceive readers or manipulate their perception in Eq 18 expressed the TWSE where c = 3.


$$R_{TWSE}=\left\{ \begin{array}{l} 0,\;if\;TW\leq c\\ 1,\;otherwise \end{array}\right.$$ (18)

**Exclamatory Tone (ET)** changes the tone of the sentence. A normal person is considered to use a few exclamatory marks in his sentences. Therefore, Exclamatory Marks (EM) are determined as threshold limit (EM = 3) is applied to determine the legitimacy of the review calculated as in Eq 19.


$$\mathrm{ET}=\left\{ \begin{array}{l} 0,\;if\;EM\leq c\\ 1,\;if\;EM>c \end{array}\right.$$ (19)

**Reviewer gives (good, bad, average sentiment)** (**GBAS):** Although this is a new feature, which is never utilized earlier however, in this work decided to test this feature for its effect detection. This a feature is binary feature, which checks for either reviewer always gives a single type of review or not. In case, reviewer gives a single type of review than there are chances of being spam showed as in Eq 20. G indicates the Binary variable indicating good sentiment. B indicates the binary variable indicating bad sentiment.


$$\mathrm{GBAS}=\left\{ \begin{array}{l} 0,\;if\neg (G\;XOR\;B)\;AND\neg (G\;XOR\;A\\ 1,\;if(G\;XOR\;B)\;OR\;(G\;XOR\;A) \end{array}\right.$$ (20)

**Reviewer gives (good and average sentiment) (GAS):** This feature is tested to check its effect on detection. This feature is binary feature, which checks for either reviewer always gives good and average review or not. In case, reviewer gets a true than there are chances of being spam showed as in Eq 21.


$$\mathrm{GAS}=\left\{ \begin{array}{l} 0,\;if\;G\;AND\;A\\ 1,\;if\neg (G\;AND\;A) \end{array}\right.$$ (21)

**Reviewer gives (bad and average sentiment) (BAS):** This feature is tested to check its effect detection showed as in Eq 22. This feature is binary feature, which checks for either reviewer always gives bad and average features or not. In case, reviewer gets a true than there are chances of being spam.


$$\mathrm{BAS}=\left\{ \begin{array}{l} 0,\;if\;B\;AND\;A\\ 1,\;if\neg (B\;AND\;A) \end{array}\right.$$ (22)

**Reviewer give (good and bad sentiment) GBS:** This feature is tested to check its effect detection. This feature is binary feature, which checks for either reviewer always gives good and bad features or not. In case, reviewer gets a true than there are less chances of being spam showed as in Eq 23.


$$\mathrm{GBS}=\left\{ \begin{array}{l} 0,\;if\;G\;AND\;B\\ 1,\;if\neg (G\;AND\;B) \end{array}\right.$$ (23)

**Percentage Of Positive Opinion Words (PPW):** Calculate the percentage of positive sentiment words or expressions within each review calculating the following Eq 24. *OW* Presents the count of opinion words in the review text and *tnr* present the total number of reviews.


$$F_{PPW}=\frac{Number\;of+iv\;OW}{tnr}\times 100$$ (24)

**Percentage Negative Opinion Words (PNOW):** Calculate the percentage of negative sentiment words or expressions within each review calculating the following Eq 25.


$$F_{PNW}=\frac{Number\;of-iv\;OW}{tnr}\times 100$$ (25)

**Percentage of numeric used (PN):** are often posted in reviews either in case of contact no (if legitimate) or some accumulative information. Therefore, Ratio of numeric is accumulated in this feature. Then this ratio is used to check against a certain threshold to get check the legitimacy of the review. The threshold limit of numeric is set as 15% of legitimate users calculating the following Eq 26. N presents the number of numeric used.


$$\mathrm{PN}=\left\{ \begin{array}{ll} Legitimate, & if\frac{N}{T}\leq threshold\\ Suspicious/Spam, & if\frac{N}{T}>threshold \end{array}\right.$$ (26)

**Percent of Capital words (PCW):** Ratio of capital letter is accumulated in this feature. Then this ratio is used to check against a certain threshold to get check the legitimacy of the review. The threshold limit is set of 20% capital letters as legitimate calculated using Eq 27 and Eq 28. Here N_capital_ referred count of words all capital letters in the text and N_total_ referred the total number of words in the text.


$$P_{\mathrm{capital}}=\frac{N_{\mathrm{capital}}}{N_{\mathrm{total}}}\times 100$$ (27)

$$\mathrm{PCW}=\left\{ \begin{array}{ll} \mathrm{Legitimate}, & \mathrm{if}\;P_{\mathrm{capital}}\leq th\\ \mathrm{Suspicious}/\mathrm{Spam}, & \mathrm{if}\;P_{\mathrm{capital}}>th \end{array}\right.$$ (28)

**All capital word used in review:** This is an important feature so in this research those reviews which consist of all capital letters were not included as original. This is due to the fact that most of the bot’s post in capital letters as in Eq 29.


$$R^{\prime }=\{r\in R|\mathrm{isUppercase}(r)=0\}$$ (29)

*R*′ = The set of filtered reviews (excluding those in all caps).

isUppercase(*r*) is a function that returns 1 if the review *r* consists of all uppercase letters and 0 otherwise.

**Name of the brand mentioned:** This feature checks for name of brand mentioned or not. If the only one and same brand name is mentioned which is being reviewed then the review is considered legitimate else spam showed as in Eq 20.


$$\mathrm{BM}=\left\{ \begin{array}{ll} 0, & if\;BM\;and\;RB=NB.\\ 1, & if\;BM\;and\;RB\neq NB\\ Not\;Determined, & if\;BM=0 \end{array}\right.$$ (30)

Brand Mentioned (BM) = Binary variable (1 if the brand name is mentioned, 0 if not mentioned).

Reviewed Brand (RB) = Name of the brand being (NB) reviewed.

**Fig 2:**
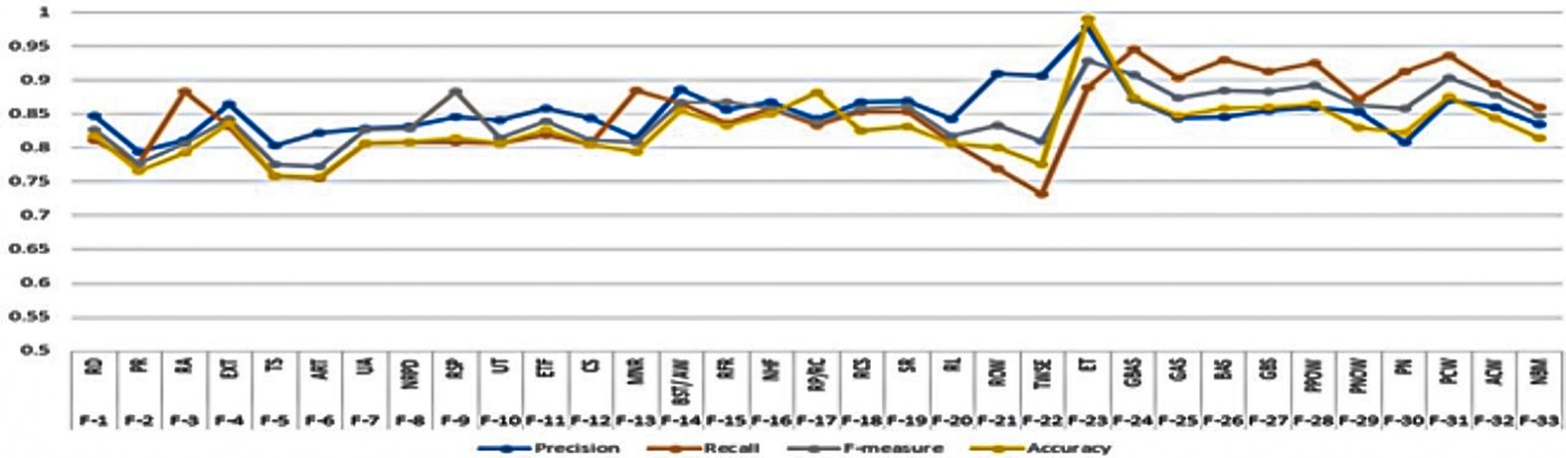
Evaluation of individual features using CRF.

The review features are normalized, and then the results are evaluated for each individual feature one by one using a CRF classifier. Random Fields with Conditions (CRF) is a model for sequence data like the Maximum Entropy Markov Model (MEMM) in that it is a discriminant model. It does this by simulating how each state depends on the whole sequence of inputs. In contrast to MEMM, CRF uses a global normalizer to deal with the problem of label bias. The results are obtained and showed in Fig 2 from CRF and stored for later comparison.

### Experimentation setup

The experiments were carried out using Python 3.7, which provided a robust foundation for our machine learning (ML) and deep learning (DL) models. We utilized NLTK 3.0 for text processing and tokenization to facilitate data normalization, while Pandas 1.2.3 allowed efficient data manipulation and transformation. Keras 2.4.3 supported the design and training of deep neural networks, and Sci-kit Learn 0.24.1 enabled the implementation of various ML methods for analyzing spammer behavior features. The experiments were executed on a Windows 10 operating system with an Intel Core i7-9700K CPU, 16 GB of RAM.

### Selection of high-performance feature sets

In the next phase, two different algorithms PCA and XGB are used to find the best-resulted features. The best-reported features set by the XGB and PCA algorithm are given in Tables 4 and 5, while. A set of features from XGB and PCA are evaluated for their accuracy using RF (Random Forest) and SVM (support vector machines), respectively. Both feature sets are evaluated to find the comparatively better feature in terms of accuracy as reported by the classifier (RF and SVM). The accuracy of both correlated feature sets is compared. It is observed that the set reported by XGB results in high accuracy. This feature set is used to train various deep learning algorithms in the third and final phase of the behavior-based spam detection framework. Various DL algorithms, including LSTM, Bi-LSTM, GRU, Bi-GRU, CNN, Char Base CNN, and CLSTM are trained using the feature set reported by the XGB method. In the end, a performance evaluation of DL methods is performed to find the most suitable DL classifier with the feature set and linguistics features to obtain maximum accuracy of spam detection.

**Table 5:** Feature selection using XGB.

Features Selection Using XGB(FSU-XGB)
(FSU-XGB) = (PCW), (ACW), (ET), (RD), (GAS), (GBAS), (BAS), (NHF), (RP/RC), (AW), (EXT)

The high-performance features are selected using the XGB [16] and PCA [19] algorithms. Both algorithms reported different features. PCA is a way to reduce the number of dimensions The steps are defined using the following equation. Derived features consist of F1, F2, F3, F4… FM are N × 1 vectors Here M = 33

**Mean Feature Vector Calculation:** The mean feature vector $\bar{F}$ computed by taking the average of all the M derived feature vectors Fi. This process involves summing up all the individual feature vectors and dividing the sum by the total number of feature vectors. By doing so, using the Eq 31 obtain a representative feature vector that captures the central tendency of the dataset.


$$\bar{F}=\frac{1}{M}\sum_{i=1}^{M}F_{i}$$ (31)

Centre the features by subtracting the mean feature vector a $\bar{F}$ from each derived feature vector *F*_*i*_.


$$\emptyset_{i}=F_{i}-\bar{F}$$ (32)

**Covariance Matrix Calculate:** Matrix A = [∅_1_∅_2_………∅_*M*_] (N×*M matrix*) constructed and calculate the covariance matrix C using Eq 33:

$$C=\frac{1}{M}\sum_{n=1}^{M}\emptyset_{n}\emptyset_{n}^{T}=\frac{1}{M}AA^{T}$$ (33)

Calculate the eigenvalues of the *λ*_1_>*λ*_2_>⋯……….*λ*_*N*_ of the covariance matrix *C*.

**Linear Conversion for Feature Reduction:** Perform feature reduction using the first $\bar{K}$ eigenvectors (≪K≪N) to represent the data in a reduced-dimensional space showed as in Eq 34:

$$\bar{F}=\sum_{i=1}^{K}b_{i}u_{i}\;where\;b_{i}=\frac{(F-\bar{F}).u_{i}}{u_{i}.u_{i}}$$ (34)

**Transformation Matrix:** Represent the process of feature reduction as a linear transformation using in Eq 35.


$$\left[b_{1},b_{2},\ldots ,b_{k}]=[u_{1}^{T},u_{2}^{T}\ldots ,u_{K}^{T}](F-\bar{F})=U^{T}(F-\bar{F}\right)$$ (35)

Using the above Eqs 31 to 35 of PCA the set of selected features as shown in Table 6 and scoring of these features are showed in Table 8. On the other hand, XGB (listed in Table 7) uses important ratings for each feature after creating boosted trees using the gradient boost method to prevent the problem associated with over-fitting. Finally, the tree provides the final target.

**Table 6:** Feature selection using PCA.

Features Selection Using PCA(FSU-PCA)
(FSU-PCA) = (RD), (EXT), (AW), (NRPD), (NHF), (UT), (RP/RC), (CS), (SR), (RL), (CRS), (ROW), (PN), (NBM), (PCW), (ACW), (ET), (GBAS), (GAS), (BAS), (GBS), (PPOW), (TWSE)

**Table 7:** Feature scoring using XGB.

F_ID	Extracted features	feature Scoring using XGB
F-1	RD	0.942
F-2	PR	0.761
F-3	RA	0.638
F-4	EXT	0.851
F-5	TS	0.645
F-6	ART	0.781
F-7	UA	0.638
F-8	NRPD	0.690
F-9	RSP	0.638
F-10	UT	0.638
F-11	ETF	0.638
F-12	CS	0.818
F-13	MNR	0.510
F-14	BST/ AW	0.839
F-15	RFR	0.630
F-16	NHF	0.970
F-17	RP/RC	0.943
F-18	RCS	0.530
F-19	SR	0.680
F-20	RL	0.730
F-21	ROW	0.763
F-22	TWSE	0.521
F-23	ET	0.946
F-24	GBAS	0.846
F-25	GAS	0.896
F-26	BAS	0.913
F-27	GBS	0.625
F-28	PPOW	0.753
F-29	PNOW	0.632
F-30	PN	0.690
F-31	PCW	0.920
F-32	ACW	0.923
F-33	NBM	0.800

**XGB** does offer feature importance scores that served as a valuable guide in the process of selecting pertinent features. These following steps provide a comprehensive overview of the mathematical procedures employed in the computation of feature importance scores utilizing the Gini index within the XGBoost framework.

**Gini Impurity for Each Feature Split:** Calculate the Gini Impurity for each feature split to quantify the level of impurity in a given set of samples. The scale ranges from 0 (pure) to 0.5 (completely impure). The Gini Impurity for a node is calculated using the Eq 36

$$G=1-\sum_{j=1}^{C}{\left(p_{ij}\right)}^{2}$$ (36)

Where *C* is the number of classes, and *p*_*ij*_ is the proportion of samples of class *j* in the node.

**Calculate the Gini Gain for Each Feature Split**: Gini Gain (*GG*) is the reduction in impurity achieved by splitting a node based on a specific feature showed in Eq 37. It’s calculated by subtracting the weighted sum of Gini impurities of child nodes from the Gini impurity of the parent node:

$$GG=G_{\mathrm{parent}}-\sum_{i=1}^{2}\frac{N_{i}}{N}G_{{\mathrm{child}}_{i}}$$ (37)

Where *N*_*i*_ is the number of samples in the *i*-th child node, *N* is the total number of samples, *G*_parent_ is the Gini impurity of the parent node, and $G_{{\mathrm{child}}_{i}}$ is the Gini impurity of the *i*-th child node.

**Calculate Feature Importance Score:** The feature importance score (FIS_*i*_) for each feature is calculated by summing up the Gini gains for all splits where that feature is used showed as in Eq 38:

$${FIS}_{i}=\sum_{\mathrm{all}\;\mathrm{splits}\;\mathrm{using}\;\mathrm{feature}\;i}GG$$ (38)

high score features are selected for model training. Both Principal Component Analysis (PCA) and XGBoost (XGB) yielded high-performance features vectors, which are detailed in Table 5 for feature selection using XGB and Table 6 for feature selection using PCA.

### Selected features

It is observed that PCA reported a set of twenty-three high-performance features (FSU-PCA) while XGB reported a set of eleven high-performance features (FSU-XGB). These identified feature sets are then evaluated to find their performance.

### Evaluation of the set of high-performance features

The feature sets derived from Principal Component Analysis (PCA) and Extreme Gradient Boosting (XGB) were utilized for spam review classification. The feature sets selected for improving the accuracy and effectiveness of spam review classification models were obtained through Principal Component Analysis (FSU-PCA) and XGBoost (FSU-XGB). The performance of the PCA-derived feature set and the XGB selected feature set was evaluated and compared to accurately identify and classify spam reviews. This study aimed to determine which feature set, based on extraction method, produced better results in terms accuracy for spam classification.

### Linguistic features

Linguistic analysis involves examining the linguistic features of the comments text to identify patterns that distinguish between legitimate and spam content. The text undergoes various prepossessing stages, including the elimination of hashtags, HTML tags, diacritics, and other extraneous symbols. These procedures are carried out to purify the test data. Additionally, primary texts and HTML tags undergo preprocessing. A distinct module has been created utilizing the functionalities of the Natural Language Tool Kit (NLTK) and Sci-Kit Learn. Linguistic features encompass various linguistic characteristics extracted from text data, including but not limited to stemming, N-gram analysis, and word2vec embeddings. These features play a crucial role in spam detection by capturing subtle language patterns indicative of spam content. Stemming reduces words to their root form, aiding in identifying recurring linguistic patterns. N-gram analysis evaluates sequences of words or characters, discerning patterns in spam text structure. Additionally, word2vec embeddings represent words as dense vectors, capturing semantic relationships and aiding in contextual understanding. By incorporating these linguistic features, the proposed model gains deeper insights into textual content, enhancing its ability to differentiate between spam and legitimate text effectively. For text processing, word embedding is an essential representation. In this way, each word is treated as if it were a token. Each word in the text is represented as a 300-dimensional or word vector. It is possible to capture the semantic relationship between individual words in this dispersed form. The sentence`s word vectors have now been contextualized using CNN or LSTM. In the CNN or LSTM, contextual information is stored instead of words, which varies from traditional word vector encoding. Dense layers are used to pass the final reduction acquired in this contextual vector. Each word indicates a time step and the label that goes with it. It is done in terms of forecasting each stage`s language id. In dataset “X_SB” be the matrix representing the Spammer Behavior features, where each row corresponds to a review and each column represents a specific spammer behavior feature. Let “X_L” be the matrix representing the linguistic features, where each row corresponds to a review and each column represents a specific linguistic feature. y be the vector of labels indicating whether each review is spam “1” or not spam “0”.

**Feature Engineering**: For features extracting each review in dataset DS, extract the spammer behavior features which consist two sets (FSU-XGB) (feature selected using XGB) and (FSU-PCA) (feature selected using PCA). Let’s denote the set of spammer behavior features as S_i(FSU-XGB) and S_i(FSU-PCA) and collectively denoted(X_SB). For each comment in dataset DL, extract the linguistic features. Let’s denote the set of linguistic features for text as X_L. S_i(FSU-PCA)" and "S_i(FSU-XGB)" are two different sets of spammer behavior features that will serve as inputs for separate algorithms, alongside linguistic features but collectively discussed as X_SB.

**Preprocessing**: By applying feature scaling to spammer behavior features and using word embeddings for linguistic features, it leveraged the strengths of each technique to enhance the representation and capture important patterns within both types of features.

**a) Feature Scaling**

Applying feature scaling to the spammer behavior features (X_SB) using standardization

Mean Calculation is performed by calculating the mean (μ) for each feature in the spammer behavior features matrix X_SB.

$$\mu_{i}=\frac{sum(X\_SB[:,i])}{N}$$ (39)

Where:

- μi represents the mean of the i^th^ feature.

X_SB[:, i] represents the ith column of the spammer behavior features matrix.

N represents the total number of samples in the dataset.

**b) Standard Deviation Calculation**:

Calculate the standard deviation (σ) for each feature in the spammer behavior features matrix X_SB.


$$\sigma_{i}=\frac{sqrt{\left(sum((X\_SB[:,i]-\mu_{i}\right)}^{\wedge }2)}{N}$$ (40)

σi represents the standard deviation of the ith feature.

X_SB[:, i] represents the ith column of the spammer behavior features matrix. μi represents the mean of the ith feature. N represents the total number of samples text in the dataset.

**c) Transforming Features**Standardization is obtained by Transform each feature in the spammer behavior features matrix X_SB to have zero mean and unit variance using the standardization formula in Eq 13.

$$X\_SB\_scaled[:,i]=\frac{\left(X\_s[:,i]-\mu_{i}\right)}{\sigma_{i}}$$ (41)

X_SB_scaled[:, i] represents the i^th^ column of the scaled spammer behavior features matrix. X_SB[:, i] represents the ith column of the spammer behavior features matrix. μ_i_ represents the mean of the ith feature. σ_i_ represents the standard deviation of the ith feature.

**Feature Fusion:** X_SB and X_L be spammer and linguistic features respectively for each review in dataset. Concatenation is performed. Eq 42 concatenate the spammer behavior features and linguistic features into a single feature vector for each review:


X_Fusion=[X_SB,X_L] (42)

The resulting unified representation

X_Fusion=f(n_samples,n_Features_SB+n_Features_L) (43)

In Eq 43 presents the n_samples are the number of reviews and n_Features_SB and n_Features_L are the number of spammer behavior and linguistic features, respectively.

**Weighted Combinations** are designed by Assigning weights w_s to the spammer behavior features and weights w_l to the linguistic features based on their relative importance. In Eq (46) showed the Multiply each feature in X_SB by its corresponding weight. Using the Eq 46 multiply each feature in X_L by its corresponding weight. The Sum up the weighted features to create a Fusions Unified (FU) representation as showed in Eq 47 and Eq 48.


X_SB_weighted(FSU_XGB)=X_SB*w_s (44)

X_SB_weighted(FSU_PCA)=X_SB*w_s (45)

X_L_weighted=X_L*w_l (46)

X_FU(PCA)=X_SB_weighted(FSU_PCA)+X_L_weighted (47)

X_FU(XGB)=X_SB_weighted(XGB)+X_L_weighted (48)

**Feature Interaction** is performed by feeding both X_SB and X_L separately as inputs to the proposed behavior-oriented deep learning model for spam classification (given in Fig 1) is implemented with various classification algorithms. The purpose was to evaluate the classifiers to achieve high classification with the best behavior feature (identified earlier).

### Implementation of model for spam classification

Feature During the classifier evaluation, the text classification results are based on the set (FSU-XGB) (feature selected using XGB) and are evaluated after comparing the classification results of various algorithms for their performance evaluation matrices. Various deep learning methods include Machine learning methods CRF, RF and SVM while deep learning methods include LSTM, Bi-LSTM, GRU, Bi-GRU, CNN, Char Base CNN, CLSTM used. The research evaluates various deep learning methods and proposed the SD-FSL-CLSTM frame work for spam detectionTwo different data set divisions are used in the training and testing phase for each deep learning algorithm. First, the algorithm is evaluated using 75:25 division ratio and then the same algorithm is evaluated using 85:15 division ratio of data set. Variations in the division of the data set are performed to check the impacts of training and testing data set ratio on the accuracy of the spam classification.

The combined input matrix is defined as in Eq (49):

$$X=\left[\begin{array}{c} X_{SB}\\ X_{L} \end{array}\right]$$ (49)

where: *X*_*SB*_∈ℝ^*m*×*n*^ is the matrix of spammer behavior features. *X*_*L*_∈ℝ^*p*×*q*^ is the matrix of linguistic features.

**Model Architecture:** The architecture of the proposed model represented as in Eq (50):

$$Y=f(W_{1}\cdot X_{SB}+W_{2}\cdot X_{L}+b)$$ (50)

where: *Y* is the output representing the classification results (spam or not spam). *W*_1_ and *W*_2_ are weight matrices corresponding to *X*_*SB*_ and *X*_*L*_.

- *b* is the bias term.
- *f* is the activation function (e.g., ReLU, Sigmoid) applied to introduce non-linearity.

Hyperparameters for the LSTM, Bi-LSTM, GRU, Bi-GRU, CNN, Char Base CNN, CLSTM used as.

Batch size: B = 64

Learning rate: α = 0.001

Dropout rate: d = 0.2

Epochs: E = 20

**A. LSTM Model Architecture:** is defined using the Eq (51)
$$h_{t}=\mathrm{LSTM}(X_{t};W_{LSTM},b_{LSTM})$$ (51)
  where *h*_*t*_ is the hidden state at time *t*, *W*_*LSTM*_ are the weights, and *b*_*LSTM*_ is the bias.
**B. Bi-LSTM Model Architecture:** is defined using the Eq (52)
$$h_{t}=\mathrm{Bi}‐\mathrm{LSTM}(X_{t};W_{Bi-LSTM},b_{Bi-LSTM})$$ (52)
**C. GRU Model Architecture:** is defined using the Eq (53)
$$h_{t}=\mathrm{GRU}(X_{t};W_{GRU},b_{GRU})$$ (53)
**D. CNN Model Architecture:** is defined using the Eq (54)
S(i,j)=(X*W)(i,j)+b (54)
  where *S*(*i*, *j*) is the output feature map after convolution, *X* is the input, and *W* is the convolution filter and the Hyperparameters are listed below.
  Filter size: *K* = 64
  Kernel size: 3×3
  Batch size: *B* = 64
  Learning rate: *α* = 0.001
  Epochs: *E* = 20
**E. Proposed SD-FSL-CLSTM Model Architecture:** is defined using the Eq (55)
$$Y=f(\mathrm{LSTM}(X_{SB};W_{LSTM},b_{LSTM})+\mathrm{CNN}(X_{L};W_{CNN},b_{CNN}))$$ (55)
  Hyperparameters for the Proposed SD-FSL-CLSTM Model is listed below
  Batch size: *B* = 32
  Learning rate: *α* = 0.001
  Dropout rate: *d* = 0.3
  Epochs: *E* = 25

### Hyperparameter Tuning Methodology

Hyperparameters were optimized using grid search defined mathematically as in Eq (56)

$$\mathrm{Opt}=\underset{\left(\alpha ,B,d\right)}{\mathrm{argmax}}\;\mathrm{Accuracy}(X,Y)$$ (56)

where Accuracy(*X*, *Y*) is the accuracy function evaluated on the validation set.

## Results and discussion

Results are inferred after the experiment to evaluate the Machine Learning and Deep Learning method for spam detection using a behavior-oriented method. Fig 1 showed the evaluation of individual features using the CRF algorithm. The conditional random field (CRF) method is used to label tokens in a sequence, and CRF focuses on ranking tokens and, thus, features in the text. It is a probabilistic graphical model that can be used to describe sequential data, such as the labels of words in a phrase. The model can also be used to determine the odds. CRF uses a set of feature functions to determine what each word in a sentence is about. These functions are designed to work with each other. During the training of the model, CRF figures out the weights of the different feature functions in a way that will make the labels in the training data appear more often this study proposed a deep learning-based spam detection framework using spammer behavior features and linguistics features of a text. This study uses the Amazon data set to evaluate the feature-oriented behavior model.

The proposed method consisted of two different steps. In the first step, each feature evaluated for its real-time contribution to classification. During this step, a total of 33 features are derived and evaluated using the CRF algorithm. CRF is composed of its feature functions. The feature functions in this study consider the current position within the sequence to determine the real value. The way in which the feature functions operate ultimately determines the resulting real value. The CRF is first evaluated for its accuracy with various extracted features. It is observed that the CRF achieved the highest accuracy of 0.991 when the exclamatory tone is selected as a feature. Other features that reported highest accuracy included Percent of Capital words (0.875) and Reviewer gives (good, bad, average sentiment) (0.874). While the CRF resulted with a minimum accuracy of.757 when reviewer review time is selected as a feature. Other features that reported lowest accuracy of classification included Time Series, Window Size (total duration in days) (0.759) and Rating Abuse (RA) (0.792). The results describe an average accuracy of 75.295, considered too low for classification. Therefore, it is recommended not to use CRF for spammer detection due to its low accuracy. Behavior-oriented classification is dependent upon feature selection. Therefore, it is necessary to find the best features. Feature selection is performed using two methods: first is XGB listed in Table 7 and second is PCA given in Table 8. The objective was to find the best features of the machine learning methods.

**Table 8:** Feature scoring using PCA.

F_ID	Extracted features	feature Scoring using PCA
F-1	RD	0.900
F-2	PR	0.601
F-3	RA	0.625
F-4	EXT	0.890
F-5	TS	0.645
F-6	ART	0.525
F-7	UA	0.638
F-8	NRPD	0.756
F-9	RSP	0.638
F-10	UT	0.640
F-11	ETF	0.520
F-12	CS	0.918
F-13	MNR	0.510
F-14	BST/ AW	0.890
F-15	RFR	0.500
F-16	NHF	0.995
F-17	RP/RC	0.954
F-18	RCS	0.735
F-19	SR	0.795
F-20	RL	0.835
F-21	ROW	0.763
F-22	TWSE	0.721
F-23	ET	0.950
F-24	GBAS	0.850
F-25	GAS	0.799
F-26	BAS	0.899
F-27	GBS	0.725
F-28	PPOW	0.799
F-29	PNOW	0.632
F-30	PN	0.501
F-31	PCW	0.560
F-32	ACW	0.620
F-33	NBM	0.630

XGB reported a set of 11 high performance features and X**_**FU(XGB) matrix is created which resulted in an average accuracy of 96% when evaluated with the Random Forest algorithm (RF). On the other hand, PCA reported a feature of 23 fesatures and with the help of theses feature X_FU(PCA) matrix is created these two matrices are fusion of spammer behavior and linguistics features as showed in Eq 47 and Eq 48.

**Fig 3:**
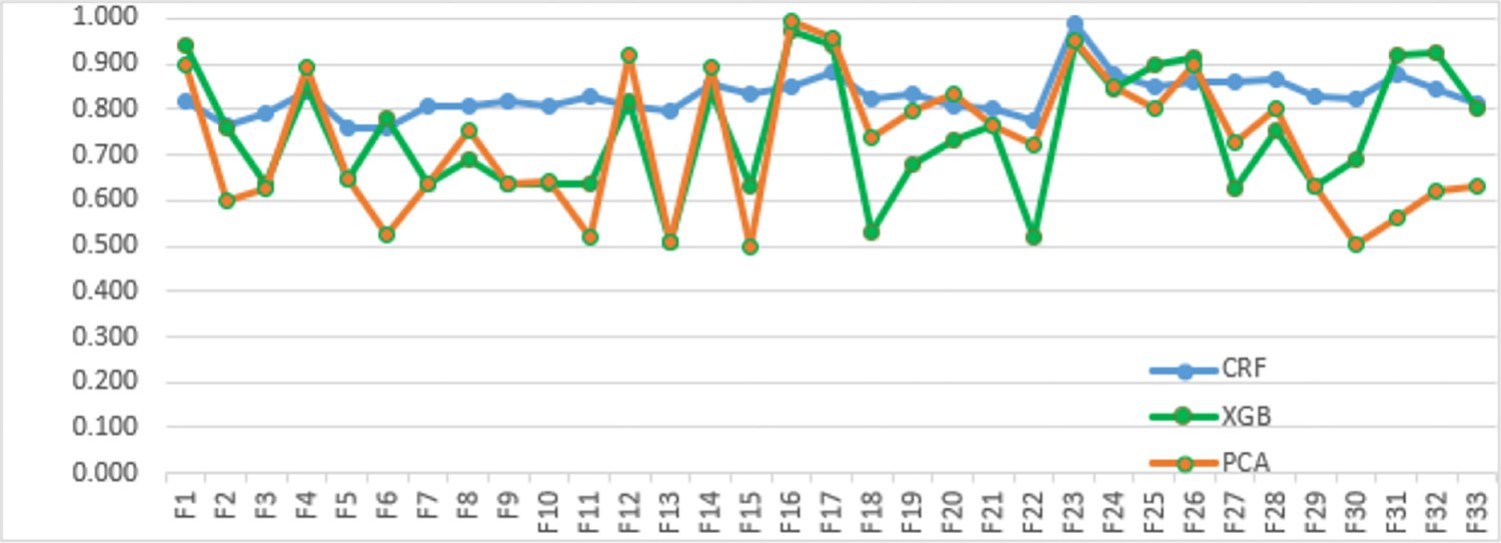
Feature scoring using CRF PCA, XGB.

That reported average accuracy of 90% as shown in Table 9 when evaluated using the RF algorithm. The following Fig 3 visualization provides an extended view of the feature scoring, showcasing the results obtained after the implementation of Conditional Random Fields (CRF), Principal Component Analysis (PCA), and XGBoost (XGB).

**Table 9:** Evaluation matrices of the proposed approach.

Classifier	Technique	X_FU(XGB) Accuracy	X_FU(PCA) Accuracy
NB	Bigram	91%	88%
LR	Uni+Bigram	88%	82%
SVM	Unigram	90%	87%
RF	Uni+Bigram+Trigram	96%	90%

X_FU(PCA) = Fusion unified matrix using PCA selected feature (SB)

X_FU(XGB) = Fusion unified matrix using XGB selected feature (SB)

Therefore, this work considered X_FU(XGB) and X_FU(PCA) as candidate features for the evaluation of the deep learning method. The selected high-performance features are used to train deep learning algorithms to finally select a robust and accurate method for behavior-oriented spam classification. As shown in Table 10 seven different deep learning algorithms trained using data set variations (85:15 & 75:25) for train and test data set ratio.

**Table 10:** Results on evaluation metrics of proposed deep learning methods on linguistic features.

Classifier	Train, Test, Ratio	X_L Accuracy	X_FU(XGB) Accuracy	X_FU(PCA) Accuracy
LSTM	85: 15	91.28	92.48	91.17
	75: 25	90.85	91.96	89.12
Bi-LSTM	85: 15	93.83	94.23	88.15
	75: 25	91.63	93.86	86.75
GRU	85: 15	92.88	93.13	88.56
	75: 25	92.15	91.98	87.12
Bi-GRU	85: 15	94.86	95.98	86.85
	75: 25	94.25	94.75	84.74
CNN	85: 15	95.86	94.58	92.46
	75: 25	95.75	93.95	91.05
Char Base CNN	85: 15	96.60	97.87	96.56
	75: 25	95.95	96.01	95.29
^**a**^ **SD-FSL-CLSTM Proposed**	85: 15	97.57	98.66	97.25
	75: 25	97.46	97.89	96.12

^**a**^ **SD-FSL-CLSTM** = Spam Detection–Fusion of Spammer and Linguistic Features long short-term memory networks

The results described that SD-FSL-C LSTM obtained the highest accuracy of 97.57 which is obtained with an 85:15 data set division ratio. The same algorithm achieved a bit low accuracy (97.46) when the data set division ratio was 75:25. Char-CNN obtained the second highest accuracy of 96.75 obtained with an 85:15 data set division ratio. The same algorithm achieved a bit low accuracy (95.60) when the data set division ratio was 75:25. While LSTM obtained minimum accuracy of 90.85 with 75:25 division ratio and 91.28 with 85:15 data set division. Similarly, GRU obtained a minimum accuracy of 92.15 obtained with 85:15 data set division ratios. The same algorithm achieved a bit better accuracy (94.86) when the data set division ratio was 75:25. It is observed that 85:15 divisions of the data are better in terms of accuracy and precision enhancement. All the deep learning algorithms reported better on the division of data set with 85:15 ratios. The results are presented in Table 10. The following table show the that includes the requested methods, algorithms, accuracy, features used. The Table 11 highlights how the proposed SD-FSL-C LSTM model performs better compared to recent works, with a notable accuracy of **97.57%** on amazon, and 95.86% on YelpChi using spammer and linguistic features. Table 11 presents a comprehensive comparison between the results of the proposed approach and the current state-of-the-art methods. It outlines key performance indicators, including accuracy, offering a clear evaluation of how the proposed methodology compares to existing techniques in the domain

**Table 11:** The proposed approach and state-of-the-art study results comparison.

References	Dataset	Algorithm	Accuracy
[57]	Amazon	BERT	0.8896%
[58]	Amazon	BERT	92%
[59]	Amazon	SVM	88%
[9]	Amazon	NB, RF, LR, SVM	93.1%
[55]	YelpChi, Amazon	Neural Network + Reinforcement Learning	97% (Amazon), 94.23% (YelpChi)
(Proposed) SD-FSL-C LSTM	Amazon YelpChi	LSTM+CNN	97.57%(Amazon), 95.86% (YelpChi)

## Conclusion

This study proposes a deep learning-based spam detection method and evaluates various spammer behavior features to select high-performance features with maximized accuracy of text classifications. PCA and XGB method is used for feature selection from spammer behaviors and then evaluated with NB, LR, SVM, and RF classifiers. The average accuracy for PCA based selected features was 91% on RF. Therefore, for feature selection, XGB is recommended for its enhanced classification accuracy because it increases the accuracy by 5% when used on RF. During the Experimentation with the CRF algorithm, Exclamatory Tone and Percent of Capital words are identified as features with the highest accuracy of text classification. Feeding the separately processed X_SB and X_L as inputs to the CLSTM model does not involve any additional computations that scale with the size of the dataset. The purposed Model SD-FSL-CLSTM, in fusion with spammer behavior and linguistic features, offers a promising approach for effective spam review detection leading to improved performance or a more comprehensive understanding of the data. Moreover, from the Experimentation, it is concluded that the feature selection impacts the classification accuracy and the data set division ratio. Accuracy is enhanced with more training data in the case of deep learning methods.

## Future work

Further research could be conducted to improve the performance of the spam detection model by incorporating additional linguistic and behavioral features. One potential area of focus could be the use of machine learning techniques to automatically identify and extract relevant features from the text of comments. Another possibility could be to incorporate other feature selection techniques like using swarm optimization algorithms related to the user’s past behavior, such as the history of their comments or the products they have previously reviewed. It would also be useful to explore the use of transfer learning, in which a model trained on one domain is fine-tuned for use in a different domain. One potential challenge in using linguistic and behavioral features for spam detection is the need to handle multiple languages. To address this issue, it may be necessary to develop language-specific models or to use machine translation to translate reviews into a common language. It would also be interesting to investigate the use of unsupervised learning techniques, such as clustering or density estimation, to identify patterns in the data that may be indicative of spam. The use of real-time analysis could be implemented to detect spam as it occurs, rather than relying on historical data. Finally, further work could be done to examine the ethical and social implications of spam detection, including the potential for unintended consequences or discrimination.

## Supporting information

S1 Data (RAR)
